# Sophisticated Gene Regulation for a Complex Physiological System: The Role of Non-coding RNAs in Photoreceptor Cells

**DOI:** 10.3389/fcell.2020.629158

**Published:** 2021-01-18

**Authors:** Sabrina Carrella, Sandro Banfi, Marianthi Karali

**Affiliations:** ^1^Telethon Institute of Genetics and Medicine (TIGEM), Pozzuoli, Italy; ^2^Medical Genetics, Department of Precision Medicine, University of Campania “Luigi Vanvitelli”, Naples, Italy; ^3^Eye Clinic, Multidisciplinary Department of Medical, Surgical and Dental Sciences, University of Campania “Luigi Vanvitelli”, Naples, Italy

**Keywords:** photoreceptors, cones, rods, retina, non-coding RNAs, microRNAs, lncRNAs, circRNAs

## Abstract

Photoreceptors (PRs) are specialized neuroepithelial cells of the retina responsible for sensory transduction of light stimuli. In the highly structured vertebrate retina, PRs have a highly polarized modular structure to accommodate the demanding processes of phototransduction and the visual cycle. Because of their function, PRs are exposed to continuous cellular stress. PRs are therefore under pressure to maintain their function in defiance of constant environmental perturbation, besides being part of a highly sophisticated developmental process. All this translates into the need for tightly regulated and responsive molecular mechanisms that can reinforce transcriptional programs. It is commonly accepted that regulatory non-coding RNAs (ncRNAs), and in particular microRNAs (miRNAs), are not only involved but indeed central in conferring robustness and accuracy to developmental and physiological processes. Here we integrate recent findings on the role of regulatory ncRNAs (e.g., miRNAs, lncRNAs, circular RNAs, and antisense RNAs), and of their contribution to PR pathophysiology. We also outline the therapeutic implications of translational studies that harness ncRNAs to prevent PR degeneration and promote their survival and function.

## Introduction

**Figure 1:**
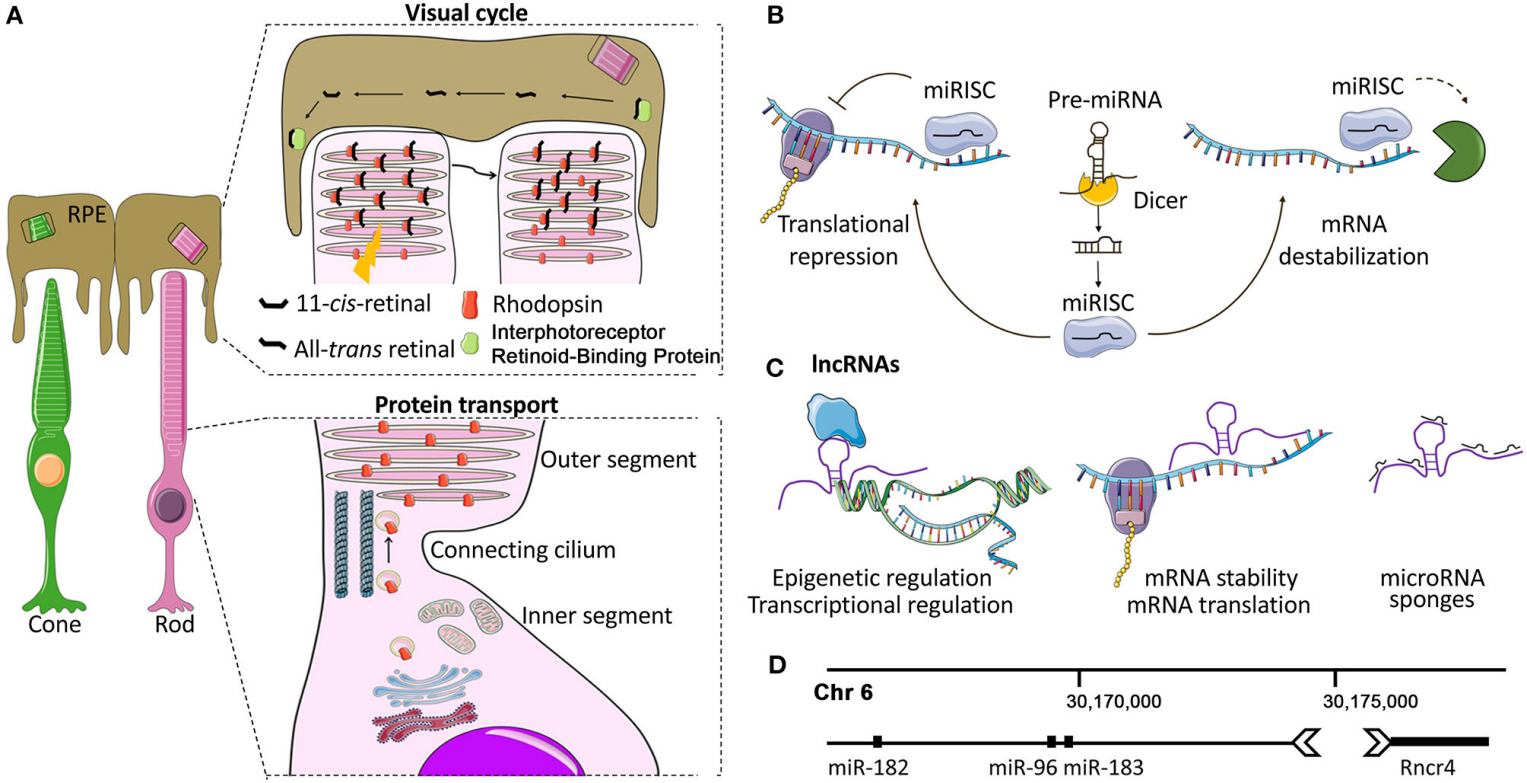
Photoreceptors and non-coding RNA mechanisms of gene regulation. **(A)** Photoreceptor structure and function. Each photoreceptor (PR) comprises a synaptic terminal that partakes in interactions with second-order retinal cells, an inner segment (IS) that contains cell organelles, and a connecting cilium that joins the IS with the outer segment (OS). The OS is a finely articulated sensory cilium (Pearring et al., 2013). It envelopes a series of stacked lamellae (membranous discs) that contain the light-sensitive opsin proteins (e.g., Rhodopsin and cone opsins) which convert photons into electrochemical signals via phototransduction cascade (Pearring et al., 2013). The PR OS physically interacts with the adjacent retinal pigment epithelium (RPE). This dynamic interplay between PRs and RPE is essential for the phagocytosis of the shed OS (Kevany and Palczewski, 2010), the transport of the circulating glucose from the choroid (Ait-Ali et al., 2015), the maintenance of ionic and osmotic balance as well as for phototransduction and visual cycle. **(B)** Overview of miRNA biogenesis and mode of action. Mature miRNAs are 20-25-nt long ncRNAs. In the canonical biogenesis pathway, they are enzymatically released from longer precursors with cleavage steps that take place in the nucleus and in the cytoplasm and are controlled by the Drosha/DGCR8 and the Dicer-TRBP complex, respectively (Krol et al., 2010b; Reh and Hindges, 2018). The mature miRNA, loaded into the RNA-induced silencing complex (RISC), recognizes and binds to complementary sequences of mRNA targets, mostly localized to 3′ untranslated regions (3′ UTR), inducing gene silencing (Krol et al., 2010b; Reh and Hindges, 2018). **(C)** Overview of lncRNA biogenesis and mode of action. LncRNAs interact with a range of cellular molecules such as other RNAs (miRNAs, mRNAs), DNA, and proteins or peptides. These interactions represent the lncRNA interactome and play a variety of roles in cell development and the pathogenesis of various diseases (Kazimierczyk et al., 2020). LncRNAs can act by different mechanisms, still not completely elucidated: (a) nuclear LncRNAs can act as epigenetic regulators, recruiting activator/repressor chromatin modifying complexes on their target promoters; (b) they can regulate transcription by guiding or preventing the recruitment of TFs on the promoters of their targets or on other active chromatin sites; (c) cytoplasmic lncRNAs may be endowed with post-transcriptional activity by regulating mRNA stability and translation, modulating mRNA degradation, and by acting as miRNA sponges (Gourvest et al., 2019). **(D)** miR-183/96/182-Rncr4: an example of crosstalk between lncRNAs and miRNAs in PRs. Graphical representation of the region of mouse chromosome 6 where the lncRNA Rncr4 (retinal non-coding RNA 4) and the miR-183/96/182 cluster are localized. Rncr4 is expressed in PRs and is transcribed in the opposite direction of the pri-miR-183/96/182. Its expression stimulates pri-miR-183/96/182 processing. Their regulatory crosstalk is important for the correct formation of the PR and inner nuclear layers (Krol et al., 2015). Details on the molecular mechanism are provided in the text.

Photoreceptors (PRs) are specialized neuronal cells adapted to the conversion of light stimuli into electrical signals. Rods are sensitive to dim light and essential for night vision, while cones enable high acuity daylight vision and color perception (Figure 1A). PRs are among the cell types of our organism that are exposed to high levels of cellular stressors (e.g., light exposure and sustained protein synthesis). First, PRs have an elevated metabolic demand due to the high rates of ion transport, opsin protein turnover, and trafficking from inner (IS) to outer segments (OS) (Figure 1A). This high energy requirement, which is almost double for a cone compared to a rod cell (Ingram et al., 2020), renders PRs vulnerable to fluctuations in energy flow. The high rate of protein synthesis poses a challenge to proteostasis (Athanasiou et al., 2013) and may expose PRs to endoplasmic reticulum stress. Second, the inevitable photo-oxidative stress requires continual clearance of Reactive Oxygen Species (Organisciak and Vaughan, 2010). In addition, PRs need to remove the toxic retinoid byproducts of the visual cycle (Figure 1A).

The structural and functional integrity of PRs is crucial for vision. Defects or mere fluctuations in any of the key molecular processes involved in PR homeostasis (e.g., energy metabolism including retinal blood flow, lipofuscin clearance, protein sorting, vesicle, and intra-flagellar transport, etc.) due to genetic changes or environmental insults can lead to PR dysfunction, cell death, and ultimately to blindness (Pearring et al., 2013). Because of their high metabolic activity, PR dysfunction is often the only phenotypic read-out of mutations in genes with ubiquitous expression, such as in the case of key ciliary genes (e.g., *CEP290* and *RPGR*) or splicing factors (e.g., *PRPF31*).

Non-coding RNAs (ncRNAs) are established as key regulators of several developmental and physiological processes. Here we discuss the role of regulatory ncRNAs in conferring robustness to PR development and function. Because of space limitations, ncRNAs that impact PR homeostasis through non-cell-autonomous processes (e.g., expression in other retinal cell-types or extracellular vesicles; Xu et al., 2019; Morris et al., 2020; Wooff et al., 2020) are not discussed in this mini-review.

## miRNA-Mediated Regulation is Essential for Photoreceptor Maturation, Function, and Survival

MiRNAs have emerged as an intriguing class of regulatory ncRNAs because of their powerful and well-conserved mechanism of sequence-specific post-transcriptional gene regulation (Bartel, 2018; Figure 1B). In particular, each miRNA is predicted to recognize on average 200 mRNA targets (Bartel, 2018), allowing for a pleiotropic fine-tuning of correlated pathways that confers robustness to biological processes (Ebert and Sharp, 2012). This multiple targeting, together with sequence similarities (i.e., miRNA families) and functional redundancy complicate the study of miRNA function.

An important piece of information to start deciphering miRNA function in the retina came from studies which defined miRNA expression in this tissue (Karali and Banfi, 2019). PR-expressed miRNAs were identified either by RNA *in situ* hybridization approaches (Kapsimali et al., 2007; Karali et al., 2007, 2010; Xu et al., 2007; Zhuang et al., 2020) or by small RNA-Seq analysis of sorted cone cells (Busskamp et al., 2014). More detailed information on the miRNA complement of PRs may derive from single-cell based sequencing approaches adapted to small RNA analysis, as already reported for mRNAs (Macosko et al., 2015; Lukowski et al., 2019; Peng et al., 2019). Besides their PR-specific expression, altered miRNA expression profiles in retinal degeneration models, even at early pre-symptomatic stages, underscored miRNA significance in PR pathophysiology (Loscher et al., 2007, 2008; Genini et al., 2014; Saxena et al., 2015; Palfi et al., 2016; Anasagasti et al., 2018).

### Photoreceptor-Specific Depletion of miRNA Biogenesis Impacts Photoreceptor Morphogenesis and Function

Functional studies based on global disruption of miRNA processing in post-mitotic PR cells, mostly through generation of *Drosha/Dgcr8* or *Dicer1* conditional knockout (cKO) mouse models, demonstrated the importance of miRNAs for PR function and survival. In one example, miRNA depletion in mature rod PRs of *Dicer1* conditional-knockout (cKO) mice led to an early-onset severe retinal degeneration (Sundermeier et al., 2014). The initial disorganization of rod OS in cKO mice was followed by an almost complete loss of PR nuclei in the ONL, depletion of the visual chromophore and severely reduced rod-mediated scotopic electroretinography (ERG). However, the cKO retinas did not display primary defects in phototransduction or in the visual cycle, suggesting that observed degeneration could be due to defects in rod maturation or homeostasis.

MiRNA depletion in adult differentiated cones following conditional *Dgcr8* ablation, led to loss of cone OS and impaired cone-mediated responses both in ERG as well as in *ex vivo* whole-cell patch clamp tests (Busskamp et al., 2014). However, cone numbers did not diminish significantly, suggesting there was no direct impact on cone cell death. Adeno-associated viral vector (AAV)-based reintroduction of the PR-enriched miRNAs miR-182 and miR-183 was sufficient to prevent cone OS loss *in vivo*, while expression of the miR-183/96/182 cluster in three-dimensional optic cup cultures from mouse embryonic stem cells induced the formation of additional PR structural components (i.e., IS, CC, and OS).

More recently, the conditional loss of *Dicer1* in developing cones was shown to cause early onset cone dystrophy (Aldunate et al., 2019). Already at 3 weeks of age, Dicer1-depleted cones had shortened and abnormal OS. This structural disorganization gradually led to loss of cone PRs and reduced photopic vision. Conversely, rod survival and function were not affected.

Considering the well-established mutual interdependence between the retina and the retinal pigment epithelium (RPE), it is indisputable that proper miRNA expression in the latter tissue is also essential for PR morphogenesis and function. This was demonstrated with the conditional deletion of *Dicer1* in the developing RPE which dramatically impacted on OS formation and PR survival in a non-cell-autonomous manner (Ohana et al., 2015).

### Functional Significance of Specific miRNAs in PRs

Loss-of-function studies of specific miRNAs have provided further insights on miRNA function in PRs (Table 1). A well-studied example is the light-regulated polycistronic miRNA cluster composed of miR-183/96/182 (Krol et al., 2010a). Collectively, this miRNA cluster accounts for almost half of the total miRNA population in human retina samples. MiR-182 and miR-183 are the most highly expressed retinal miRNAs (Karali et al., 2016), and are highly enriched in PRs (Karali et al., 2007, 2010; Xu et al., 2007; Zhu et al., 2011; Lumayag et al., 2013; Busskamp et al., 2014; Sundermeier et al., 2014). Sponge-mediated inactivation of the miR-183/96/182 cluster in mouse mature rods did not induce apparent morphological or functional defects under normal light conditions. Instead, it sensitized retinas to bright light-induced retinal degeneration, suggesting this cluster has a protective role under stress conditions (Zhu et al., 2011). On the contrary, constitutive gene-trap-based inactivation of the miR-183/96/182 cluster in mice not only increased susceptibility to light damage but led to an early-onset progressive retinal degeneration associated with defects in the phototransduction cascade and the PRs' synaptic connectivity (Lumayag et al., 2013). In agreement, miR-183/96/182 *null* mice, obtained by targeted recombination, display profound defects in vision and other sensory functions, mainly due to impaired terminal differentiation of sensory neurons. PR ciliogenesis and OS formation was delayed leading to early-onset degeneration (Fan et al., 2017). Finally, the double miR-183 and miR-96 KO mouse had defects in cone polarization and OS morphogenesis that gradually led to PR degeneration, underscoring their importance for PR maturation and maintenance (Xiang et al., 2017). The authors proposed that the effect of miR-183 and miR-96 on PR development is mediated through the regulation of the taurine transporter *Slc6a6*. Recently, miR-183 KO mice presented mild yet progressive defects in ERG responses (Zhang et al., 2020a). Additionally, miR-182-depleted mice showed a thinner IS/OS layer, a progressive reduction of scotopic and photopic ERG responses and increased sensitivity to light-damage compared to control littermates (Wu et al., 2019). In contrast, a study assessing the potential redundancy of the cluster members reported the absence of retinal defects in single, double, and triple mir-183/96/182 mutant zebrafish (Fogerty et al., 2019).

**Table 1:** Summary of representative non-coding RNAs with a role in photoreceptor function and maintenance.

**ncRNAs**	**Function and main features**	**Therapeutic perspectives**	**References**
**miRNAs**			
miR-182, miR-183, miR-96	Their expression is light-regulated; inactivation is linked to PR and synaptic transmission dysfunction, impairment of cone maturation and maintenance, with progressive degeneration; increased expression preserves cone OS in *Dicer1-*ablated PRs		Krol et al., 2010a; Zhu et al., 2011; Lumayag et al., 2013; Busskamp et al., 2014; Fan et al., 2017; Xiang et al., 2017; Wu et al., 2019; Peskova et al., 2020; Zhang et al., 2020a
miR-124	Predominantly localized to PR IS; promotes maturation and survival of cone photoreceptors by targeting *Lhx2*	Intravitreal miR-124 administration decreases microglia infiltration and PR cell death in mice after light-induced damage	Karali et al., 2007; Sanuki et al., 2011; Chu-Tan et al., 2018
miR-204, miR-211	Silencing in medaka-fish has a negative impact on PR maintenance and function, leading to increased apoptosis Genetic inactivation of miR-211 in mice leads to progressive cone dystrophy, cone loss, and alterations in visual function. A point mutation in the miR-204 mature sequence associated with retinal dystrophy and coloboma in humans	AAV-mediated subretinal delivery in mouse models of RP promotes PR survival and preserves retinal function through a synergistic effect on innate immunity, inflammatory response and cell death	Conte et al., 2015; Barbato et al., 2017; Karali et al., 2020
miR-6937-5p	Upregulated at early stages of retinal degeneration in *rd10* mice	AAV-mediated inhibition in *rd10* mice delays PR demise and vision loss	Anasagasti et al., 2018, 2020
**LncRNAs**			
*TUG1*	Involved in PR development and survival; loss-of-function is associated with defects in PR differentiation, structural defects of the OS and increased apoptosis		Young et al., 2005
*Vax2os1*	Controls cell cycle progression and proliferation of PR progenitors; increased expression delays PR differentiation and enhances apoptosis		Alfano et al., 2005; Meola et al., 2012
*MEG3*	Significantly upregulated in light-induced retinal degeneration	*MEG3* silencing protects against light-induced retinal degeneration; regulates PR apoptosis by preventing p53 degradation	Wang et al., 2012; Zhou et al., 2012; Zhu et al., 2018
*MALAT1*	*MALAT1* expression is lost in rod PRs with putative degeneration, suggesting its involvement in rod PR survival and retinal preservation	Intravitreal injection of *MALAT1*-siRNA in mice with diabetes-induced and in oxygen-induced retinopathy reduced secondary PR loss	Lukowski et al., 2019; Wang et al., 2020; Zhang et al., 2020b
*RNCR4* (BB283400)	Regulates proper thickness of PR and inner nuclear layers by regulating pri-miR-183/96/182 processing		Krol et al., 2015
*circ-Tulp4*	Acts as a miR-26a/671/204 sponge; AAV-circ-Tulp4-shRNA-treated retinas show severe PR degeneration		Chen et al., 2020

*PRs, Photoreceptors; OS, Outer Segment; IS, Inner Segment; AAV, adeno-associated viral vector; RP, Retinitis Pigmentosa*.

Discrepancies in the phenotypic severity among the above-mentioned models may stem from differences in the methodology, developmental timing, and cell-type specificity of the cluster ablation. Nevertheless, the phenotypes are consistent with the role of the miR-183/96/182 cluster in PR differentiation and synaptic connectivity, signal transduction, transmembrane transport, cell-adhesion, regulation of circadian rhythm and apoptosis (Xu et al., 2007; Dambal et al., 2015; Palfi et al., 2016). Moreover, several mRNA targets that partake in these processes were shown to be regulated by the 183/96/182 cluster members such as *Crb1* (Krol et al., 2015), *Mitf, Adcy6* (Xu et al., 2007), *Rnf217* (Xiang et al., 2017; Zhang et al., 2020a), *Rac1, Slc6a9* (Palfi et al., 2016), *Slc1a1* (Krol et al., 2010a), *Slc6a6* (Xiang et al., 2017), and *Casp2* (Zhu et al., 2011).

Another miRNA that contributes to PR survival is miR-124, an abundant neuronal-specific miRNA expressed in all neural cells of the retina (Kapsimali et al., 2007; Karali et al., 2007, 2011, 2016; Liu et al., 2011; Sanuki et al., 2011). In adult PRs, miR-124 is predominantly localized in the IS (Karali et al., 2007; Sanuki et al., 2011). Upon degeneration induced by photo-oxidative damage, miR-124 aberrantly redistributed from PRs to the inner retina (Chu-Tan et al., 2018). This mislocalization of miR-124 was likely mediated by extracellular vesicles, as it was impaired following exosome-depletion (Wooff et al., 2020). MiR-124 depletion in a mouse KO for *Rncr3*, its main host gene, resulted in cone mislocalization and demise. Rod PRs were not significantly affected indicating that miR-124 is important primarily for cone survival by targeting *Lhx2* (Sanuki et al., 2011).

The miR-204/211 family is expressed in diverse ocular tissues consistent with its pleiotropic role in eye development (Shiels, 2020). MiR-204 and miR-211 are intragenic miRNAs transcribed from introns of the *Trpm3* and *Trpm1* genes, respectively (Barbato et al., 2017; Shiels, 2020). In the posterior eye, miR-204 is strongly expressed in the RPE and INL (Karali et al., 2007, 2011, 2016) but also detectable in PRs (Conte et al., 2015). Although PRs are not the primary site of miR-204 expression, morpholino-mediated knockdown of miR-204 adversely impacted PR maintenance and function in medaka-fish (Conte et al., 2015). Similarly, the targeted inactivation of miR-211 caused progressive cone dystrophy characterized by reduced cone-elicited ERG responses and cell density in mouse. Transcriptome analysis suggested that miR-211 can impact retinal metabolism by controlling genes involved in glucose and lipid metabolism (Barbato et al., 2017). The higher metabolic demand of cones (Cheng et al., 2020) that renders them susceptible to energy fluctuations, could explain why cones (rather than rods) were predominantly affected by miR-211 loss (Barbato et al., 2017). Likewise, the rapid light-regulated turnover of miR-204/211 in the murine retina, similar to that of the miR-183/96/182 cluster, has been postulated to facilitate activity-dependent expression changes in neuronal cells (Krol et al., 2010a). Remarkably, a heterozygous point mutation within the seed region of miR-204 segregated with a dominant retinal dystrophy characterized by severe PR loss and diminished ERG responses, presumably through a gain-of-function mechanism (Conte et al., 2015).

## Role of Specific lncRNAs in Photoreceptors

Long non-coding RNAs (lncRNAs) are a class of transcripts longer than 200 nucleotides with limited protein-coding potential. They exceed mRNAs in quantity and participate in various biological processes and functions across different cell types (Blackshaw et al., 2004; Briggs et al., 2015; Quek et al., 2015; Clark and Blackshaw, 2017; Kopp and Mendell, 2018). LncRNAs can act as decoys of transcription factors (TF) and miRNAs (Hansen et al., 2013), interact with chromatin modifiers to regulate epigenetic states (Rinn and Chang, 2012), or participate in nuclear topological organization (Ip and Nakagawa, 2012) and in protein-complex scaffolding (Ribeiro et al., 2018; Figure 1C). LncRNAs have recently been found to contribute to PR function (Table 1).

The availability of high-throughput sequencing approaches has facilitated the comprehensive and unbiased identification of lncRNAs in the retina. Palczewski and colleagues identified a group of 18 highly conserved large intergenic non-coding RNAs (lincRNAs) in the retina, some of which localized to specific retinal layers, especially to the PR layer (Mustafi et al., 2013).

One of the first lncRNAs studied in the retina was the *Taurine Upregulated Gene 1 (TUG1)*. Loss-of-function of *TUG1* in the developing retina showed its implication in normal PR development, by acting on transcriptional regulation of PR-specific genes through a yet unknown mechanism. *TUG1* absence in the newborn retina led to structural defects in PR OS accompanied by increased apoptosis and expression changes of key TFs and marker genes of differentiated PRs (Young et al., 2005). A recent study reported the dynamics of the non-coding transcriptome in developing and mature PRs, focusing on rod-enriched lncRNAs controlled by the rod differentiation factor Nrl. The dynamic expression of the non-coding transcriptome during rod maturation is consistent with its functional relevance in the morphogenesis of OS membrane discs and synapse formation (Zelinger et al., 2017). The authors also identified a functional role of antisense RNAs (asRNAs), a subclass of lncRNAs that originate from the opposite strand of 20–40% of protein-coding genes, in PR formation. AsRNAs were identified as putative regulators of eye development because of their overlap with the mRNAs of TFs known to play key roles in vertebrate eye development (Alfano et al., 2005). Among them *Vax2os1*, where “os” stands for “opposite strand,” shows an expression confined to the ventral portion of the eye and is predominantly detected in the layers where PR progenitors and differentiated cells reside. Consistent with this expression pattern, *Vax2os1* overexpression in the retina impaired cell cycle progression of PR progenitors and delayed PR differentiation. At later developmental stages, this perturbation led to increased apoptosis in the PR layer (Meola et al., 2012).

Recently, the *Maternally Expressed 3 (MEG3)* was shown to be involved in the pathogenesis of light-induced retinal degeneration. *MEG3* expression is significantly upregulated upon light insult whereas its silencing protects against light-induced retinal degeneration *in vivo* and *in vitro* by decreasing caspase 3/7 activity and proapoptotic protein (Bax) levels while upregulating antiapoptotic protein (Bcl-2) expression. Mechanistically, *MEG3* regulates PR apoptosis by acting as a p53 decoy, preventing MDM2-mediated p53 degradation (Wang et al., 2012; Zhou et al., 2012; Zhu et al., 2018).

Another lncRNA strongly upregulated in stress conditions is *MALAT1*, expressed in all retinal layers (Yao et al., 2016). *MALAT1* expression in rod PRs was reduced upon longer post-mortem times, prompting the authors to suggest that such downregulation may be linked to an early stage of rod PR degeneration (Lukowski et al., 2019).

An interesting example of crosstalk between lncRNAs and miRNAs in PR cells is represented by the lncRNA *Rncr4* (retinal non-coding RNA 4) and the miR-183/96/182 cluster (Krol et al., 2015; Figure 1D). *Rncr4* is expressed in maturing PRs in the opposite direction to the pri-miR-183/96/182. Its expression stimulates pri-miR-183/96/182 processing by acting on the activity of the DEAD-box RNA helicase/ATPase Ddx3x, an inhibitor of pri-miR-183/96/182 maturation in early postnatal PRs. Alteration of the timing of miR-183/96/182 formation led to early accumulation of mature miR-183/96/182 and caused profound irregularities in the thickness of the PR and inner nuclear layers (Krol et al., 2015).

A novel class of ncRNAs with tissue- and stage-specific expression patterns are the circular RNAs (circRNAs) whose circular characteristic confers increased stability compared to linear transcripts (Rybak-Wolf et al., 2015; Han et al., 2017). CircRNAs display high expression, evolutionary conservation, specificity of action and stability. They are involved in numerous biological processes and are differentially expressed in various diseases (Ghosal et al., 2013; Lukiw, 2013; Li et al., 2015; Peng et al., 2015; Zhao and Shen, 2017), including diabetic retinopathy, highlighting their potential as biomarkers and as predictors of response to treatments (Li et al., 2015; Chen et al., 2016; Shan et al., 2017; Zhang et al., 2017, 2018). Transcriptome profiling of circRNAs at five different developmental stages in wild-type and degenerating retinas (*rd8* mouse model; Mehalow et al., 2003) identified circRNAs with a dynamic and strong expression pattern. These circRNAs were found to target miRNAs in the retina, acting as competitive endogenous RNAs (ceRNAs). Specifically, *Cdr1as* acted as miR-204/677/378 sponge, *circHipk2* as a miR-124 sponge, *circTulp4* as a miR-26a/671/204 sponge, and *circAnkib1* as a miR-195a sponge. Functional analysis of *circ-Tulp4* revealed that its reduction leads to downregulation of miR-204-5p and miR-26a-5p targets (i.e., *Meis2, Cdh2, Mitf*, and *Pde4b*) which are major players in retinal development and function. Accordingly, AAV-*circTulp4*-shRNA-treated retinas showed severe PR degeneration with attenuated scotopic and photopic ERG responses confirming that *circTulp4* is indispensable for proper retinal function. The authors observed that the levels of some circRNAs, but not those of their corresponding linear transcripts, increased before disease onset in the retina of rd8 mice (Chen et al., 2020). More comprehensive transcriptome studies extended to additional models are needed to support the hypothesis that circRNA expression alterations may be early markers of PR degeneration.

## Perspectives

Advancements in understanding the role of ncRNAs in PR development and function prompted the design of new strategies to promote PR survival (Table 1). Specifically, miRNA modulation can impact simultaneously on several biological processes that exacerbate PR degeneration as shown in the following examples of miRNA-based therapeutics. First, intravitreal delivery of miR-124 mimics reduced microglia infiltration and PR cell death following light-induced damage in mice, possibly by modulating *ccl2* levels (Chu-Tan et al., 2018). In a similar approach, the subretinal delivery of AAV-miR-204 in a mouse model of dominant RP (i.e., the transgenic *RHO*-P347S) promoted PR survival and preserved retinal function through synergistic effects on innate immunity, inflammatory responses, and cell death (Karali et al., 2020). miR-204 exerted this protective effect, at least partially, by downregulating the expression of *Siglec1* and *Xaf1* (Karali et al., 2020). Finally, AAV-mediated inhibition of miR-6937-5p in *rd10* mice delayed PR and vision loss (Anasagasti et al., 2020). This miRNA was upregulated at early stages of retinal degeneration in *rd10* mice (Anasagasti et al., 2018). Recently, intravitreal injection of *MALAT1*-siRNA in diabetes-induced mice reduced the functional and morphological damage of rods and cones, indicating its protective, likely indirect, effect against secondary PR loss (Zhang et al., 2020b). Similarly, *MALAT1* increase in oxygen-induced retinopathy was counteracted by intravitreal injection of MALAT1-siRNA, which significantly improved the retinal phenotype (Wang et al., 2020). Although these results highlight *MALAT1* silencing as a possible therapeutic strategy for different forms of retinopathy, the direct functional role of *MALAT1* in PRs remains unclear, especially in light of the results linking its reduction to PR degeneration (Lukowski et al., 2019). The discrepancy between the above observations warrant further investigation aimed at a better clarification of the role of *MALAT1* in the different retinal cell types.

These studies underscore the therapeutic potential of ncRNA-based approaches in mutation- and gene-independent approaches. Such strategies are particularly relevant for inherited retinal diseases due to their high genetic heterogeneity (Carrella et al., 2020). Moreover, they are appealing for gain-of-function and dominant negative mutations where gene supplementation is not appropriate to overturn the consequences of the genetic lesion. Similar attempts applied to non-retinal conditions yielded encouraging outcomes confirming the translational potential of ncRNA-based therapies in human disease (Hanna et al., 2019; Dammes and Peer, 2020).

## Author Contributions

SC and SB conceived the manuscript. SC and MK wrote the first draft of the manuscript. SB edited the manuscript. All authors contributed to the article and approved the submitted version.

## Conflict of Interest

The authors declare that the research was conducted in the absence of any commercial or financial relationships that could be construed as a potential conflict of interest.

## Abbreviations

AAV: adeno-associated viral vector
asRNA: antisense RNA
CC: connecting cilium
ceRNA: competitive endogenous RNA
circRNA: circular RNA
cKO: conditional knockout
CNV: choroidal neovascularization
Dgcr8: DiGeorge Critical Region 8
ERG: electroretinography
hPSCs: human pluripotent stem cells
IS: inner segment
KO: knockout
lincRNAs: large intergenic non-coding RNAs
lncRNA: long non-coding RNA
MALAT1: Metastasis Associated Lung Adenocarcinoma Transcript 1
MEG3: Maternally Expressed 3
miRNA: microRNA
ncRNA: non-coding RNA
ONL: outer nuclear layer
OS: outer segment
P: postnatal day
PRs: photoreceptors
RGC: retinal ganglion cells
RPC: retinal progenitor cells
RP: Retinitis Pigmentosa
RPE: Retinal Pigment Epithelium
TRPM1/3: transient receptor potential cation channel subfamily M member 1/3
TUG1: Taurine Upregulated Gene 1.

## References

[B1] Ait-Ali N. Fridlich R. Millet-Puel G. Clerin E. Delalande F. Jaillard C.. (2015). Rod-derived cone viability factor promotes cone survival by stimulating aerobic glycolysis. Cell 161, 817–832. 10.1016/j.cell.2015.03.023 25957687

[B2] Aldunate E. Z. Di Foggia V. Di Marco F. Hervas L. A. Ribeiro J. C. Holder D. L.. (2019). Conditional Dicer1 depletion using Chrnb4-Cre leads to cone cell death and impaired photopic vision. Sci. Rep. 9:2314. 10.1038/s41598-018-38294-9 30783126 PMC6381178

[B3] Alfano G. Vitiello C. Caccioppoli C. Caramico T. Carola A. Szego M. J.. (2005). Natural antisense transcripts associated with genes involved in eye development. Hum. Molecular Genet. 14, 913–923. 10.1093/hmg/ddi084 15703187

[B4] Anasagasti A. Ezquerra-Inchausti M. Barandika O. Munoz-Culla M. Caffarel M. M. Otaegui D.. (2018). Expression profiling analysis reveals key MicroRNA–mRNA interactions in early retinal degeneration in retinitis pigmentosa. Investig. Ophthalmol. Vis. Sci. 59, 2381–2392. 10.1167/iovs.18-24091 29847644 PMC5939684

[B5] Anasagasti A. Lara-López A. Milla-Navarro S. Escudero-Arrarás L. Rodríguez-Hidalgo M. Zabaleta N.. (2020). Inhibition of MicroRNA 6937 delays photoreceptor and vision loss in a mouse model of retinitis pigmentosa. Pharmaceutics 12:913. 10.3390/pharmaceutics12100913 32987664 PMC7598722

[B6] Athanasiou D. Aguil,à M. Bevilacqua D. Novoselov S. S. Parfitt D. A. Cheetham M. E. (2013). The cell stress machinery and retinal degeneration. FEBS Lett. 587, 2008–2017. 10.1016/j.febslet.2013.05.020 23684651 PMC4471140

[B7] Barbato S. Marrocco E. Intartaglia D. Pizzo M. Asteriti S. Naso F.. (2017). MiR-211 is essential for adult cone photoreceptor maintenance and visual function. Sci. Rep. 7:17004. 10.1038/s41598-017-17331-z 29209045 PMC5717140

[B8] Bartel D. P. (2018). Metazoan micrornas. Cell 173, 20–51. 10.1016/j.cell.2018.03.006 29570994 PMC6091663

[B9] Blackshaw S. Harpavat S. Trimarchi J. Cai L. Huang H. Kuo W. P.. (2004). Genomic analysis of mouse retinal development. PLoS Biol. 2:e247. 10.1371/journal.pbio.0020247 15226823 PMC439783

[B10] Briggs J. A. Wolvetang E. J. Mattick J. S. Rinn J. L. Barry G. (2015). Mechanisms of long non-coding RNAs in mammalian nervous system development, plasticity, disease, and evolution. Neuron 88, 861–877. 10.1016/j.neuron.2015.09.045 26637795

[B11] Busskamp V. Krol J. Nelidova D. Daum J. Szikra T. Tsuda B.. (2014). miRNAs 182 and 183 are necessary to maintain adult cone photoreceptor outer segments and visual function. Neuron 83, 586–600. 10.1016/j.neuron.2014.06.020 25002228

[B12] Carrella S. Indrieri A. Franco B. Banfi S. (2020). Mutation-independent therapies for retinal diseases: focus on gene-based approaches. Front. Neurosci. 14:1015. 10.3389/fnins.2020.588234 33071752 PMC7541846

[B13] Chen X.-J. Zhang Z.-C. Wang X.-Y. Zhao H.-Q. Li M.-L. Ma Y.. (2020). The circular RNome of developmental retina in mice. Mol. Ther. Nucleic Acids 19, 339–349. 10.1016/j.omtn.2019.11.016 31877410 PMC6938940

[B14] Chen Y. Li C. Tan C. Liu X. (2016). Circular RNAs: a new frontier in the study of human diseases. J. Med. Genet. 53, 359–365. 10.1136/jmedgenet-2016-103758 26945092

[B15] Cheng S. Y. Cipi J. Ma S. Hafler B. P. Kanadia R. N. Brush R. S.. (2020). Altered photoreceptor metabolism in mouse causes late stage age-related macular degeneration-like pathologies. Proc. Natl. Acad. Sci. U.S.A. 117, 13094–13104. 10.1073/pnas.2000339117 32434914 PMC7293639

[B16] Chu-Tan J. A. Rutar M. Saxena K. Aggio-Bruce R. Essex R. W. Valter K.. (2018). MicroRNA-124 Dysregulation is associated with retinal inflammation and photoreceptor death in the degenerating retina. Investig. Ophthalmol. Vis. Sci. 59, 4094–4105. 10.1167/iovs.18-24623 30098196 PMC11647551

[B17] Clark B. S. Blackshaw S. (2017). Understanding the role of lncRNAs in nervous system development. Adv. Exp. Med. Biol. 1008, 253–282. 10.1007/978-981-10-5203-3_9 28815543 PMC5890441

[B18] Conte I. Hadfield K. D. Barbato S. Carrella S. Pizzo M. Bhat R. S.. (2015). MiR-204 is responsible for inherited retinal dystrophy associated with ocular coloboma. Proc. Natl. Acad. Sci. U.S.A. 112, E3236–3245. 10.1073/pnas.1401464112 26056285 PMC4485104

[B19] Dambal S. Shah M. Mihelich B. Nonn L. (2015). The microRNA-183 cluster: the family that plays together stays together. Nucleic Acids Res. 43, 7173–7188. 10.1093/nar/gkv703 26170234 PMC4551935

[B20] Dammes N. Peer D. (2020). Paving the road for RNA therapeutics. Trends Pharmacol. Sci. 41, 755–775. 10.1016/j.tips.2020.08.004 32893005 PMC7470715

[B21] Ebert M. S. Sharp P. A. (2012). Roles for microRNAs in conferring robustness to biological processes. Cell 149, 515–524. 10.1016/j.cell.2012.04.005 22541426 PMC3351105

[B22] Fan J. Jia L. Li Y. Ebrahim S. May-Simera H. Wood A.. (2017). Maturation arrest in early postnatal sensory receptors by deletion of the miR-183/96/182 cluster in mouse. Proc. Natl. Acad. Sci. U.S.A. 114, E4271–E4280. 10.1073/pnas.1619442114 28484004 PMC5448201

[B23] Fogerty J. Stepanyan R. Cianciolo L. T. Tooke B. P. Perkins B. D. (2019). Genomic non-redundancy of the mir-183/96/182 cluster and its requirement for hair cell maintenance. Sci. Rep. 9:10302. 10.1038/s41598-019-46593-y 31311951 PMC6635406

[B24] Genini S. Guziewicz K. E. Beltran W. A. Aguirre G. D. (2014). Altered miRNA expression in canine retinas during normal development and in models of retinal degeneration. BMC Genomics 15:172. 10.1186/1471-2164-15-172 24581223 PMC4029133

[B25] Ghosal S. Das S. Sen R. Basak P. Chakrabarti J. (2013). Circ2Traits: a comprehensive database for circular RNA potentially associated with disease and traits. Front. Genet. 4:283. 10.3389/fgene.2013.00283 24339831 PMC3857533

[B26] Gourvest M. Brousset P. Bousquet M. (2019). Long noncoding RNAs in acute myeloid leukemia: functional characterization and clinical relevance. Cancers 11:1638. 10.3390/cancers11111638 31653018 PMC6896193

[B27] Han J. Gao L. Dong J. Bai J. Zhang M. Zheng J. (2017). The expression profile of developmental stage-dependent circular RNA in the immature rat retina. Mol. Vis. 23, 457–469. 28761319 PMC5524268

[B28] Hanna J. Hossain G. S. Kocerha J. (2019). The potential for microRNA therapeutics and clinical research. Front. Genet. 10:478. 10.3389/fgene.2019.00478 31156715 PMC6532434

[B29] Hansen T. B. Jensen T. I. Clausen B. H. Bramsen J. B. Finsen B. Damgaard C. K.. (2013). Natural RNA circles function as efficient microRNA sponges. Nature 495, 384–388. 10.1038/nature11993 23446346

[B30] Ingram N. T. Fain G. L. Sampath A. P. (2020). Elevated energy requirement of cone photoreceptors. Proc. Natl. Acad. Sci. U.S.A. 117, 19599–19603. 10.1073/pnas.2001776117 32719136 PMC7431031

[B31] Ip J. Y. Nakagawa S. (2012). Long non-coding RNAs in nuclear bodies. Develop. Growth Different. 54, 44–54. 10.1111/j.1440-169X.2011.01303.x 22070123

[B32] Kapsimali M. Kloosterman W. P. de Bruijn E. Rosa F. Plasterk R. H. Wilson S. W. (2007). MicroRNAs show a wide diversity of expression profiles in the developing and mature central nervous system. Genome Biol. 8:R173. 10.1186/gb-2007-8-8-r173 17711588 PMC2375003

[B33] Karali M. Banfi S. (2019). Non-coding RNAs in retinal development and function. Hum. Genet. 138, 957–971. 10.1007/s00439-018-1931-y 30187163

[B34] Karali M. Guadagnino I. Marrocco E. De Cegli R. Carissimo A. Pizzo M. (2020). AAV-miR-204 protects from retinal degeneration by attenuation of microglia activation and photoreceptor cell death. Mol. Ther. Nucleic Acids 19, 144–156. 10.1016/j.omtn.2019.11.005 31837604 PMC6920266

[B35] Karali M. Manfredi A. Puppo A. Marrocco E. Gargiulo A. Allocca M.. (2011). MicroRNA-restricted transgene expression in the retina. PLoS ONE 6:e22166. 10.1371/journal.pone.0022166 21818300 PMC3144214

[B36] Karali M. Peluso I. Gennarino V. A. Bilio M. Verde R. Lago G.. (2010). miRNeye: a microRNA expression atlas of the mouse eye. BMC Genomics 11:715. 10.1186/1471-2164-11-715 21171988 PMC3018480

[B37] Karali M. Peluso I. Marigo V. Banfi S. (2007). Identification and characterization of microRNAs expressed in the mouse eye. Invest. Ophthalmol. Vis. Sci. 48, 509–515. 10.1167/iovs.06-0866 17251443

[B38] Karali M. Persico M. Mutarelli M. Carissimo A. Pizzo M. Singh Marwah V.. (2016). High-resolution analysis of the human retina miRNome reveals isomiR variations and novel microRNAs. Nucleic Acids Res. 44, 1525–1540. 10.1093/nar/gkw039 26819412 PMC4770244

[B39] Kazimierczyk M. Kasprowicz M. K. Kasprzyk M. E. Wrzesinski J. (2020). Human long noncoding RNA interactome: detection, characterization and function. Int. J. Mol. Sci. 21, 1027. 10.3390/ijms21031027 32033158 PMC7037361

[B40] Kevany B. M. Palczewski K. (2010). Phagocytosis of retinal rod and cone photoreceptors. Physiology 25, 8–15. 10.1152/physiol.00038.2009 20134024 PMC2839896

[B41] Kopp F. Mendell J. T. (2018). Functional classification and experimental dissection of long noncoding RNAs. Cell 172, 393–407. 10.1016/j.cell.2018.01.011 29373828 PMC5978744

[B42] Krol J. Busskamp V. Markiewicz I. Stadler M. B. Ribi S. Richter J.. (2010a). Characterizing light-regulated retinal microRNAs reveals rapid turnover as a common property of neuronal microRNAs. Cell 141, 618–631. 10.1016/j.cell.2010.03.039 20478254

[B43] Krol J. Krol I. Alvarez C. P. P. Fiscella M. Hierlemann A. Roska B.. (2015). A network comprising short and long noncoding RNAs and RNA helicase controls mouse retina architecture. Nat. Commun. 6:7305. 10.1038/ncomms8305 26041499 PMC4468907

[B44] Krol J. Loedige I. Filipowicz W. (2010b). The widespread regulation of microRNA biogenesis, function and decay. Nat. Rev. Genet. 11, 597–610. 10.1038/nrg2843 20661255

[B45] Li Y. Zheng Q. Bao C. Li S. Guo W. Zhao J.. (2015). Circular RNA is enriched and stable in exosomes: a promising biomarker for cancer diagnosis. Cell Res. 25, 981–984. 10.1038/cr.2015.82 26138677 PMC4528056

[B46] Liu K. Liu Y. Mo W. Qiu R. Wang X. Wu J. Y.. (2011). MiR-124 regulates early neurogenesis in the optic vesicle and forebrain, targeting NeuroD1. Nucleic Acids Res. 39, 2869–2879. 10.1093/nar/gkq904 21131276 PMC3074159

[B47] Loscher C. J. Hokamp K. Kenna P. F. Ivens A. C. Humphries P. Palfi A.. (2007). Altered retinal microRNA expression profile in a mouse model of retinitis pigmentosa. Genome Biol. 8:R248. 10.1186/gb-2007-8-11-r248 18034880 PMC2258196

[B48] Loscher C. J. Hokamp K. Wilson J. H. Li T. Humphries P. Farrar G. J.. (2008). A common microRNA signature in mouse models of retinal degeneration. Exper. Eye Res. 87, 529–534. 10.1016/j.exer.2008.08.016 18834879 PMC4030402

[B49] Lukiw W. (2013). Circular RNA (circRNA) in Alzheimer's disease (AD). Front. Genet. 4:307. 10.3389/fgene.2013.00307 24427167 PMC3875874

[B50] Lukowski S. W. Lo C. Y. Sharov A. A. Nguyen Q. Fang L. Hung S. S.. (2019). A single-cell transcriptome atlas of the adult human retina. EMBO J. 38:e100811. 10.15252/embj.2018100811 31436334 PMC6745503

[B51] Lumayag S. Haldin C. E. Corbett N. J. Wahlin K. J. Cowan C. Turturro S.. (2013). Inactivation of the microRNA-183/96/182 cluster results in syndromic retinal degeneration. Proc. Natl. Acad. Sci. U.S.A. 110, E507–516. 10.1073/pnas.1212655110 23341629 PMC3568372

[B52] Macosko E. Z. Basu A. Satija R. Nemesh J. Shekhar K. Goldman M.. (2015). Highly parallel genome-wide expression profiling of individual cells using nanoliter droplets. Cell 161, 1202–1214. 10.1016/j.cell.2015.05.002 26000488 PMC4481139

[B53] Mehalow A. K. Kameya S. Smith R. S. Hawes N. L. Denegre J. M. Young J. A.. (2003). CRB1 is essential for external limiting membrane integrity and photoreceptor morphogenesis in the mammalian retina. Hum. Mol. Genet. 12, 2179–2189. 10.1093/hmg/ddg232 12915475

[B54] Meola N. Pizzo M. Alfano G. Surace E. M. Banfi S. (2012). The long noncoding RNA Vax2os1 controls the cell cycle progression of photoreceptor progenitors in the mouse retina. RNA 18, 111–123. 10.1261/rna.029454.111 22128341 PMC3261733

[B55] Morris D. R. Bounds S. E. Liu H. Ding W.-Q. Chen Y. Liu Y.. (2020). Exosomal MiRNA transfer between retinal microglia and RPE. Int. J. Mol. Sci. 21:3541. 10.3390/ijms21103541 32429541 PMC7279010

[B56] Mustafi D. Kevany B. M. Bai X. Maeda T. Sears J. E. Khalil A. M.. (2013). Evolutionarily conserved long intergenic non-coding RNAs in the eye. Hum. Mol. Genet. 22, 2992–3002. 10.1093/hmg/ddt156 23562822 PMC3699063

[B57] Ohana R. Weiman-Kelman B. Raviv S. Tamm E. R. Pasmanik-Chor M. Rinon A.. (2015). MicroRNAs are essential for differentiation of the retinal pigmented epithelium and maturation of adjacent photoreceptors. Development 142, 2487–2498. 10.1242/dev.121533 26062936

[B58] Organisciak D. T. Vaughan D. K. (2010). Retinal light damage: mechanisms and protection. Progr. Retinal Eye Res. 29, 113–134. 10.1016/j.preteyeres.2009.11.004 19951742 PMC2831109

[B59] Palfi A. Hokamp K. Hauck S. M. Vencken S. Millington-Ward S. Chadderton N.. (2016). microRNA regulatory circuits in a mouse model of inherited retinal degeneration. Sci. Rep. 6:31431. 10.1038/srep31431 27527066 PMC4985623

[B60] Pearring J. N. Salinas R. Y. Baker S. A. Arshavsky V. Y. (2013). Protein sorting, targeting and trafficking in photoreceptor cells. Prog. Retin. Eye Res. 36, 24–51. 10.1016/j.preteyeres.2013.03.002 23562855 PMC3759535

[B61] Peng L. Yuan X. Q. Li G. C. (2015). The emerging landscape of circular RNA ciRS-7 in cancer. Oncol. Rep. 33, 2669–2674. 10.3892/or.2015.3904 25873049

[B62] Peng Y.-R. Shekhar K. Yan W. Herrmann D. Sappington A. Bryman G. S.. (2019). Molecular classification and comparative taxonomics of foveal and peripheral cells in primate retina. Cell 176, 1222. e22–1237. e22. 10.1016/j.cell.2019.01.004 30712875 PMC6424338

[B63] Peskova L. D. Jurcikova T. Vanova J. Krivanek M. Capandova Z. Sramkova J.. (2020). miR-183/96/182 cluster is an important morphogenetic factor targeting PAX6 expression in differentiating human retinal organoids. Stem Cells 38, 1557–1567. 10.1002/stem.3272 32875669

[B64] Quek X. C. Thomson D. W. Maag J. L. Bartonicek N. Signal B. Clark M. B.. (2015). lncRNAdb v2. 0: expanding the reference database for functional long noncoding RNAs. Nucleic Acids Res. 43, D168-D173. 10.1093/nar/gku988 25332394 PMC4384040

[B65] Reh T. A. Hindges R. (2018). MicroRNAs in retinal development. Annu. Rev. Vis. Sci. 4, 25–44. 10.1146/annurev-vision-091517-034357 29889656

[B66] Ribeiro D. M. Zanzoni A. Cipriano A. Delli Ponti R. Spinelli L. Ballarino M.. (2018). Protein complex scaffolding predicted as a prevalent function of long non-coding RNAs. Nucleic Acids Res. 46, 917–928. 10.1093/nar/gkx1169 29165713 PMC5778612

[B67] Rinn J. L. Chang H. Y. (2012). Genome regulation by long noncoding RNAs. Ann. Rev. Biochem. 81, 145–166. 10.1146/annurev-biochem-051410-092902 22663078 PMC3858397

[B68] Rybak-Wolf A. Stottmeister C. GlaŽar P. Jens M. Pino N. Giusti S.. (2015). Circular RNAs in the mammalian brain are highly abundant, conserved, and dynamically expressed. Mol. Cell 58, 870–885. 10.1016/j.molcel.2015.03.027 25921068

[B69] Sanuki R. Onishi A. Koike C. Muramatsu R. Watanabe S. Muranishi Y.. (2011). miR-124a is required for hippocampal axogenesis and retinal cone survival through Lhx2 suppression. Nature neuroscience 14, 1125–1134. 10.1038/nn.2897 21857657

[B70] Saxena K. Rutar M. V. Provis J. M. Natoli R. C. (2015). Identification of miRNAs in a model of retinal degenerations. Investig. Ophthalmol. Vis. Sci. 56, 1820–1829. 10.1167/iovs.14-15449 25711632

[B71] Shan K. Liu C. Liu B.-H. Chen X. Dong R. Liu X.. (2017). Circular noncoding RNA HIPK3 mediates retinal vascular dysfunction in diabetes mellitus. Circulation 136, 1629–1642. 10.1161/CIRCULATIONAHA.117.029004 28860123

[B72] Shiels A. (2020). TRPM3_miR-204: a complex locus for eye development and disease. Hum. Genomics 14, 1–24. 10.1186/s40246-020-00258-4 32070426 PMC7027284

[B73] Sundermeier T. R. Zhang N. Vinberg F. Mustafi D. Kohno H. Golczak M.. (2014). DICER1 is essential for survival of postmitotic rod photoreceptor cells in mice. FASEB J. 28, 3780–3791. 10.1096/fj.14-254292 24812086 PMC4101655

[B74] Wang P. Ren Z. Sun P. (2012). Overexpression of the long non-coding RNA MEG3 impairs in vitro glioma cell proliferation. J. Cell. Biochem. 113, 1868–1874. 10.1002/jcb.24055 22234798

[B75] Wang Y. Wang X. Wang Y.-,x. Ma Y. Di Y. (2020). Effect and mechanism of the long noncoding RNA MALAT1 on retinal neovascularization in retinopathy of prematurity. Life Sci. 260:118299. 10.1016/j.lfs.2020.118299 32827542

[B76] Wooff Y. Cioanca A. V. Chu-Tan J. A. Aggio-Bruce R. Schumann U. Natoli R. (2020). Small-medium extracellular vesicles and their miRNA Cargo in retinal health and degeneration: mediators of homeostasis, and vehicles for targeted gene therapy. Front. Cell. Neuroscie. 14:160. 10.3389/fncel.2020.00160 32670023 PMC7330137

[B77] Wu K.-C. Chen X.-J. Jin G.-H. Wang X.-Y. Yang D.-D. Li Y.-P.. (2019). Deletion of miR-182 leads to retinal dysfunction in mice. Investigative ophthalmology and visual science 60, 1265–1274. 10.1167/iovs.18-24166 30924851

[B78] Xiang L. Chen X.-J. Wu K.-C. Zhang C.-J. Zhou G.-H. Lv J.-N.. (2017). miR-183/96 plays a pivotal regulatory role in mouse photoreceptor maturation and maintenance. Proc. Natl. Acad. Sci. U.S.A. 114, 6376–6381. 10.1073/pnas.1618757114 28559309 PMC5474811

[B79] Xu S. Witmer P. D. Lumayag S. Kovacs B. Valle D. (2007). MicroRNA (miRNA) transcriptome of mouse retina and identification of a sensory organ-specific miRNA cluster. J. Biol. Chem. 282, 25053–25066. 10.1074/jbc.M700501200 17597072

[B80] Xu W. Wu Y. Hu Z. Sun L. Dou G. Zhang Z.. (2019). Exosomes from microglia attenuate photoreceptor injury and neovascularization in an animal model of retinopathy of prematurity. Mol. Ther. Nucleic Acids 16, 778–790. 10.1016/j.omtn.2019.04.029 31163320 PMC6545376

[B81] Yao J. Wang X. Q. Li Y. J. Shan K. Yang H. Wang Y. N. Z. (2016). Long non-coding RNA MALAT 1 regulates retinal neurodegeneration through CREB signaling. EMBO Mol. Med. 8, 346–362. 10.15252/emmm.201505725 26964565 PMC4818754

[B82] Young T. Matsuda T. Cepko C. (2005). The noncoding RNA taurine upregulated gene 1 is required for differentiation of the murine retina. Curr. Biol. 15, 501–512. 10.1016/j.cub.2005.02.027 15797018

[B83] Zelinger L. Karakülah G. Chaitankar V. Kim J.-W. Yang H.-J. Brooks M. J.. (2017). Regulation of noncoding transcriptome in developing photoreceptors by rod differentiation factor NRL. Investig. Ophthalmol. Vis. Sci. 58, 4422–4435. 10.1167/iovs.17-21805 28863214 PMC5584472

[B84] Zhang C.-J. Xiang L. Chen X.-J. Wang X.-Y. Wu K.-C. Zhang B.-W.. (2020a). Ablation of mature miR-183 leads to retinal dysfunction in mice. Investig. Ophthalmol. Vis. Sci. 61:12. 10.1167/iovs.61.3.12 32176259 PMC7401733

[B85] Zhang S.-J. Chen X. Li C.-P. Li X.-M. Liu C. Liu B.-H.. (2017). Identification and characterization of circular RNAs as a new class of putative biomarkers in diabetes retinopathy. Investig. Ophthalmol. Vis. Sci. 58, 6500–6509. 10.1167/iovs.17-22698 29288268

[B86] Zhang Y.-L. Hu H.-Y. You Z.-P. Li B.-Y. Shi K. (2020b). Targeting long non-coding RNA MALAT1 alleviates retinal neurodegeneration in diabetic mice. Int. J. Ophthalmol. 13, 213–219. 10.18240/ijo.2020.02.03 32090029 PMC7013796

[B87] Zhang Z. Yang T. Xiao J. (2018). Circular RNAs: promising biomarkers for human diseases. EBioMedicine 34, 267–274. 10.1016/j.ebiom.2018.07.036 30078734 PMC6116471

[B88] Zhao Z.-J. Shen J. (2017). Circular RNA participates in the carcinogenesis and the malignant behavior of cancer. RNA Biol. 14, 514–521. 10.1080/15476286.2015.1122162 26649774 PMC5449088

[B89] Zhou Y. Zhang X. Klibanski A. (2012). MEG3 noncoding RNA: a tumor suppressor. J. Mol. Endocrinol. 48, R45–R53. 10.1530/JME-12-0008 22393162 PMC3738193

[B90] Zhu Q. Sun W. Okano K. Chen Y. Zhang N. Maeda T.. (2011). Sponge transgenic mouse model reveals important roles for the microRNA-183 (miR-183)/96/182 cluster in postmitotic photoreceptors of the retina. J. Biol. Chem. 286, 31749–31760. 10.1074/jbc.M111.259028 21768104 PMC3173082

[B91] Zhu Y.-X. Yao J. Liu C. Hu H.-T. Li X.-M. Ge H.-M.. (2018). Long non-coding RNA MEG3 silencing protects against light-induced retinal degeneration. Biochem. Biophys. Res. Commun. 496, 1236–1242. 10.1016/j.bbrc.2018.01.177 29409883

[B92] Zhuang P. Zhang H. Welchko R. M. Thompson R. C. Xu S. Turner D. L. (2020). Combined microRNA and mRNA detection in mammalian retinas by *in situ* hybridization chain reaction. Sci. Rep. 10:351. 10.1038/s41598-019-57194-0 31942002 PMC6962165

